# Ocrelizumab reduces thalamic volume loss in patients with RMS and PPMS

**DOI:** 10.1177/13524585221097561

**Published:** 2022-06-07

**Authors:** Douglas L Arnold, Till Sprenger, Amit Bar-Or, Jerry S Wolinsky, Ludwig Kappos, Shannon Kolind, Ulrike Bonati, Stefano Magon, Johan van Beek, Harold Koendgen, Oscar Bortolami, Corrado Bernasconi, Laura Gaetano, Anthony Traboulsee

**Affiliations:** Montreal Neurological Institute, McGill University, Montreal, QC, Canada/NeuroRx Research, Montreal, QC, Canada; Department of Neurology, DKD Helios Klinik Wiesbaden, Wiesbaden, Germany/Research Center for Clinical Neuroimmunology and Neuroscience and MS Center, Departments of Medicine, Clinical Research and Biomedical Engineering, University Hospital Basel and University of Basel, Basel, Switzerland; Department of Neurology and Center for Neuroinflammation and Experimental Therapeutics, Perelman School of Medicine, University of Pennsylvania, Philadelphia, PA, USA; McGovern Medical School, The University of Texas Health Science Center at Houston (UTHealth), Houston, TX, USA; Research Center for Clinical Neuroimmunology and Neuroscience and MS Center, Departments of Medicine, Clinical Research and Biomedical Engineering, University Hospital Basel and University of Basel, Basel, Switzerland; University of British Columbia, Vancouver, BC, Canada; Hoffmann-La Roche Ltd, Basel, Switzerland; F. Hoffmann-La Roche Ltd, Basel, Switzerland; F. Hoffmann-La Roche Ltd, Basel, Switzerland/Biogen, Baar, Switzerland; F. Hoffmann-La Roche Ltd, Basel, Switzerland/UCB Farchim SA, Bulle, Switzerland; F. Hoffmann-La Roche Ltd, Basel, Switzerland; F. Hoffmann-La Roche Ltd, Basel, Switzerland; F. Hoffmann-La Roche Ltd, Basel, Switzerland; University of British Columbia, Vancouver, BC, Canada

**Keywords:** Ocrelizumab, multiple sclerosis, thalamus, atrophy, treatment outcome

## Abstract

**Background:**

In multiple sclerosis (MS), thalamic integrity is affected directly by demyelination and neuronal loss, and indirectly by gray/white matter lesions outside the thalamus, altering thalamic neuronal projections.

**Objective:**

To assess the efficacy of ocrelizumab compared with interferon beta-1a (IFNβ1a)/placebo on thalamic volume loss and the effect of switching to ocrelizumab on volume change in the Phase III trials in relapsing MS (RMS, OPERA I/II; NCT01247324/NCT01412333) and in primary progressive MS (PPMS, ORATORIO; NCT01194570).

**Methods:**

Thalamic volume change was computed using paired Jacobian integration and analyzed using an adjusted mixed-effects repeated measurement model.

**Results:**

Over the double-blind period, ocrelizumab treatment significantly reduced thalamic volume loss with the largest effect size (Cohen’s *d*: RMS: 0.561 at week 96; PPMS: 0.427 at week 120) compared with whole brain, cortical gray matter, and white matter volume loss. At the end of up to 7 years of follow-up, patients initially randomized to ocrelizumab still showed less thalamic volume loss than those switching from IFNβ1a (*p* < 0.001) or placebo (*p* < 0.001).

**Conclusion:**

Ocrelizumab effectively reduced thalamic volume loss compared with IFNβ1a/placebo. Early treatment effects on thalamic tissue preservation persisted over time. Thalamic volume loss could be a potential sensitive marker of persisting tissue damage.

## Introduction

The thalamus harbors several relay nuclei with extensive cortical and subcortical connections. It is affected by multiple sclerosis (MS) by inflammatory-mediated demyelination,^1^ primary neurodegeneration of axons and neurons,^2^ and/or antero-/retro-grade axonal degeneration due to demyelination or transection of the tracts projecting into and out of the thalamus.^3^ Thus, change in thalamic volume may reflect not only local but also wider MS-related damage throughout the central nervous system (CNS).^4,5^

Thalamic volume loss occurs early in MS, including in patients with clinically isolated syndrome at presentation,^6^ in pediatric MS,^7^ and in radiologically isolated syndrome.^8^ Moreover, thalamic volume loss is associated with disability progression, measured by changes in Expanded Disability Status Scale (EDSS),^9–11^ and cognitive impairment.^12^

Post hoc analyses of randomized, controlled trials in relapsing MS (RMS) have shown that thalamic volume loss was reduced by disease-modifying therapies (DMTs) compared with placebo (e.g. laquinimod,^13^ fingolimod,^14^ and siponimod)^15^ or compared with an active treatment arm (such as daclizumab,^16^ and ozanimod).^17^ However, the effect of anti-CD20 therapies on thalamic volume loss has not been assessed. Therefore, we analyzed a large dataset of patients with RMS and primary progressive MS (PPMS) from three randomized controlled trials (OPERA I/II^18^ and ORATORIO)^19^ to assess the effect of ocrelizumab on thalamic volume change. Our aims were to evaluate: the efficacy of ocrelizumab during the double-blind periods (DBPs); the effects of switching to or maintaining ocrelizumab in the open-label extensions (OLEs); the similarities and differences in thalamic atrophy between patients with RMS and PPMS; and the relationship between thalamic volume, clinical outcomes, and disability progression.

## Materials and methods

### Trial design and population

OPERA I (NCT01247324), OPERA II (NCT01412333), and ORATORIO (NCT01194570) trials have been described previously.^18,19^ Briefly, OPERA I/II (hereafter referred to as OPERA) were two Phase III, multicenter, randomized, double-blind, double-dummy, interferon beta-1a (IFNβ1a) controlled trials with identical designs, in patients with RMS. Key eligibility criteria included an age of 18–55 years, MS diagnosis according to 2010 revised McDonald criteria,^20^ and screening EDSS score of 0.0–5.5. Following completion of the 96-week DBP of both trials, patients maintained or switched to ocrelizumab (IFNβ1a–ocrelizumab), given every 24 weeks. ORATORIO was an international, multicenter, Phase III, randomized, parallel-group, double-blind, placebo-controlled trial investigating efficacy and safety of ocrelizumab in patients with PPMS. Key eligibility criteria included an age of 18–55 years, a diagnosis of PPMS by the McDonald 2005 criteria,^21^ and screening EDSS score of 3.0–6.5. ORATORIO has three treatment periods: the DBP, an extended controlled period (ECP), and the OLE. The DBP lasted at least 120 weeks until a prespecified number of 12-week confirmed disability progression events on the EDSS (CDP12-EDSS) occurred. The subsequent ECP spanned from the end of the DBP to the first OLE dose of ocrelizumab for each individual. Patients entered the OLE between 144–294 weeks after randomization, where patients maintained or switched to ocrelizumab (placebo–ocrelizumab), given every 24 weeks.

The OLEs of all trials are ongoing. The data for this analysis were collected approximately to the end of 2019. By this cut-off date, patients had been exposed to ocrelizumab for up to 7 years (5 in OLE) in OPERA, and for up to 6.5 years (4 in OLE) in ORATORIO. The relevant institutional review boards/ethics committees approved the trial protocols and all patients provided written informed consent.

### MRI volume measurements

In OPERA, magnetic resonance imaging (MRI) assessments were conducted at baseline, weeks 24, 48, and 96 in the DBP, and yearly in the OLE (OLE weeks 46, 94, 142, 190, and 238). In ORATORIO, MRI scans were acquired at baseline, weeks 24, 48, and 120 in the DBP, and at baseline (OLE day 1) and yearly thereafter in the OLE (OLE weeks 48, 96, and 144). Because the timing of MRI assessments in the ORATORIO OLE differed between patients relative to randomization, OLE MRIs were categorized relative to OLE entry date.

Whole brain, thalamic volume, cortical gray, and white matter, all normalized by head size, were assessed using baseline MRIs. Relative percentage change from baseline was obtained for each subsequent visit using SIENA (Structural Image Evaluation, using Normalization, of Atrophy) for whole brain, and paired Jacobian integration^22^ for the rest. T1-weighted three-dimensional images with 3 mm slice thickness (no gap) and whole brain coverage acquired during the studies were used for those assessments.

### Statistical analysis

All analyses used the intent-to-treat (ITT) population and the OPERA trials were pooled for this analysis. Missing data were not imputed. All statistical tests were exploratory and no adjustment for multiplicity was applied. The significance level of statistical tests was set at 5%. Analyses were performed in SAS 9.4 and R version 3.6.3. Random coefficient models were analyzed on the latter environment using package LME4 version 1.1.21.

#### Association between thalamic volume and population characteristics at baseline

DBP baseline associations between normalized thalamic volume and patients’ demographics, and disease characteristics were assessed through Spearman’s correlations for continuous variables or Wilcoxon rank-sum tests for categorical variables.

#### Longitudinal evaluation of thalamic volume

Percentage change of thalamic volume from DBP baseline was computed using a mixed-effects model of repeated measures (MMRMs) including factors for time, treatment, treatment × time, treatment × baseline thalamic volume, and adjusted for baseline characteristics: that is, age; region (United States vs rest of the world (ROW)); EDSS category (<4, ⩾4); normalized thalamic volume; presence/absence of T1 Gadolinium (Gd)-enhancing lesions; and T2 lesion volume.

Moreover, to evaluate thalamic volume decline in specific situations, linear random coefficients mixed-effects models were used, in which treatment, study (for OPERA), region, time, treatment × time (for evaluating the difference in slopes), and baseline characteristics (EDSS category, age, normalized thalamic volume, presence/absence of T1 Gd-enhancing lesions, T2 lesion volume, and previous relapses on past year ⩽1 versus >1, for OPERA) were entered as fixed effects, while participant and time (study day of the assessment) were included as random intercept and slopes, respectively. Model and bootstrapped estimates have been retrieved. From random effect models annualized percent change were computed as ((adjusted slope estimate)/(adjusted mean at hypothetical baseline) × 100).

To assess the relationship between T2 lesions and thalamic volume loss, the thalamic percentage change at the end of the DBP was correlated (using Spearman correlation) with baseline T2 lesion volume and T2 lesion volume change at the end of the DBP.

#### Treatment effect size

We computed Cohen’s *d* as the between-arm difference at the last visit of the DBP (week 96 for RMS; week 120 for PPMS) divided by the adjusted standard deviation of the measurement. Estimates were obtained from the MMRM model. In addition to thalamic volume loss, we assessed Cohen’s *d* for whole brain, cortical, and white matter volume loss.^23,24^

#### RMS versus PPMS comparison

To assess differences between the RMS and PPMS populations in a pooled analysis, baseline normalized thalamic volumes were compared with an ANCOVA corrected for age, sex, disease duration, EDSS, region, presence/absence of T1 Gd-enhancing lesions, and T2 lesion volume. Thalamic volume loss rates in ocrelizumab-treated patients were compared using a random coefficients model as previously described with an additional factor for the trial.

#### Baseline thalamic volume and clinical outcomes association

Baseline relationship between thalamic volume and clinical outcomes (i.e. EDSS, Nine-Hole Peg Test (9HPT), and Timed 25-Foot Walk (T25FW)) was assessed with linear regression analyses corrected for age, sex, presence/absence of T1 Gd-enhancing lesions, and T2 lesion volume.

The association between baseline normalized thalamic volume and future disability accumulation was evaluated by Cox proportional hazard regression models adjusted for treatment group, age, sex, EDSS score, T2 lesion volume, presence/absence of T1 Gd-enhancing lesions, normalized thalamic volume, and treatment group × thalamic volume. Time to 12- and 24-week confirmed disability progression (CDP12 and CDP24, respectively) measured by EDSS (CDP12-EDSS, CDP24-EDSS); 9HPT (CDP12-9HPT, CDP24-9HPT); T25FW (CDP12-T25FW, CDP24-T25FW); and composite CDP (CCDP12, CCDP24, defined as a confirmed occurrence of an increase in EDSS score, the time to perform the T25FW of ⩾20%, or the time to complete 9HPT of ⩾20%) in the DBP were evaluated. For all clinical tests, we investigated the predictive value of baseline thalamic volume for the treatment effect by its interaction with the treatment variable. In case of a significant interaction, we assessed each arm in a separate model.

## Results

Nearly all the ITT population (99.8%) initially randomized in OPERA and ORATORIO contributed to the analysis. The demographics and disease characteristics of the ITT population at baseline are summarized in Table 1. At baseline, the normalized thalamic volume was 15.14 ± 1.93 cm^3^ in patients with RMS and 14.41 ± 1.80 cm^3^ in patients with PPMS.

**Table 1.** Baseline characteristics of patients in the ITT population.

	RMS (OPERA I/II)		PPMS (ORATORIO)	
	IFNβ1a (*n* = 829)	Ocrelizumab (*n* = 827)	Placebo (*n* = 244)	Ocrelizumab (*n* = 488)
Age, median (range), years	37 (18–55)	38 (18–56)	46 (18–56)	46 (20–56)
Female, *n* (%)	552 (66.6)	541 (65.4)	124 (50.8)	237 (48.6)
Time since MS diagnosis, median (range), years	1.7 (0.1–28.5)	1.8 (0.0–28.9)	1.3 (0.1–23.8)	1.6 (0.1–16.8)
EDSS score, median (range)	2.5 (0.0–6.0)	2.5 (0.0–6.0)	4.5 (2.5–6.5)	4.5 (2.5–7.0)
T2w lesion volume, median (range), mL	6.2 (0.0–76.1)	5.4 (0.0–96.0)	6.2 (0.0–81.1)	7.3 (0.0–90.3)
No. of T2w lesions, median (range)	42.0 (0.0–226.0)	40.0 (1.0–233.0)	43.0 (0.0–208.0)	42.0 (0.0–249.0)
Patients with T1w Gd-enhancing lesions, no./total no. (%)	327/822 (39.8)	333/818 (40.7)	60/243 (24.7)	133/484 (27.5)
Normalized brain volume, median (range), cm^3^	1504.8 (1245.9–1751.9)	1502.4 (1202.7–1761.3)	1464.5 (1216.3–1701.7)	1462.2 (1214.3–1711.1)

EDSS: Expanded Disability Status Scale; Gd: gadolinium; IFNβ1a: interferon β-1a; ITT: intent-to-treat; PPMS: primary progressive multiple sclerosis; RMS: relapsing multiple sclerosis.

## The association between thalamic volume and population characteristics at baseline

**Figure 1.**
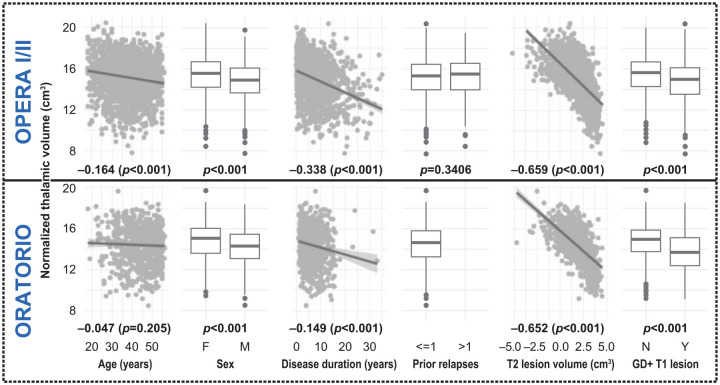
Associations between normalized thalamic volume and population characteristics at baseline. For continuous variables (i.e. age, disease duration, and T2w lesion volume), Spearman’s correlations were computed to assess the association with normalized thalamic volume. In this case, correlation coefficients and *p* values are reported. For categorical variables (i.e. sex, prior relapses, presence/absence of gadolinium-enhancing lesions), Wilcoxon rank-sum tests assessed the differences in normalized thalamic volume between groups and *p* values are reported.

The associations between normalized thalamic volume and population characteristics at baseline are summarized in Figure 1.

### Longitudinal evaluation of thalamic volume and treatment effect size

**Figure 2.**
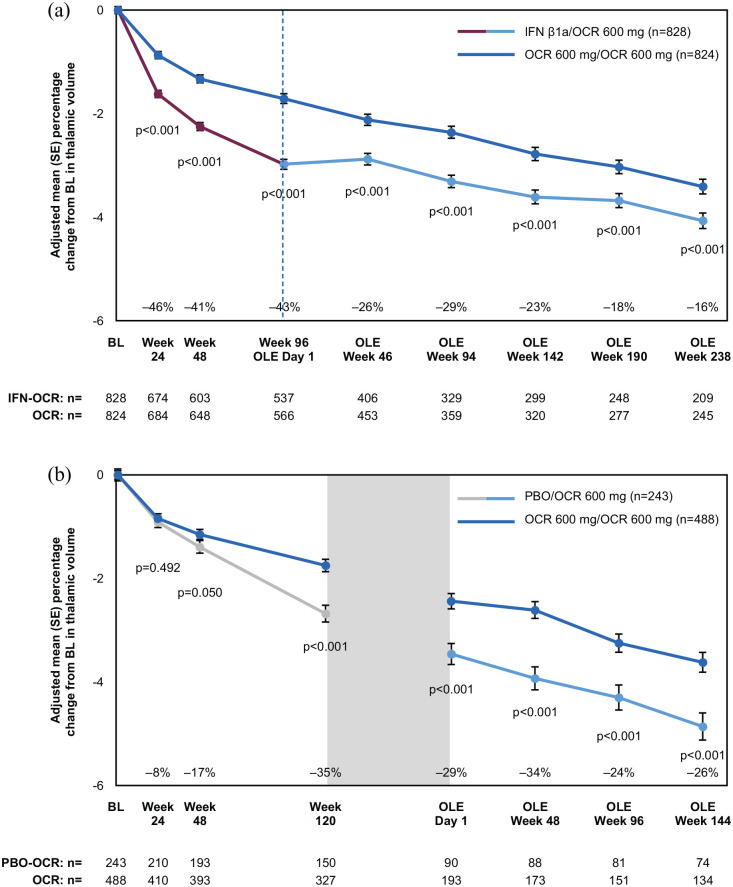
Treatment effect on thalamic volume loss over time in the RMS (a) and PPMS (b) populations. BL, baseline; IFNβ1a, interferon β-1a; OCR, ocrelizumab; OLE, open-label extension; PBO, placebo; SE, standard error. Gray box in (b) represents the transition period of PPMS patients switching from placebo to ocrelizumab and entering the OLE from the extended controlled period. Percentage reductions reported in the figure were calculated as: 100 × (ocrelizumab adjusted mean—comparator arm adjusted mean)/(comparator arm adjusted mean).

During the DBP, compared with the comparator arm, ocrelizumab progressively reduced thalamic volume loss in patients with RMS and PPMS, reaching a percentage reduction of 43% in RMS and 35% in PPMS at the end of the DBP (Figure 2). When comparing these results with the treatment effect on whole brain, cortical gray and white matter volume, the thalamus showed the largest effect size (Cohen’s *d*: RMS: 0.320, 0.383, 0.159, vs 0.561 for the thalamus; PPMS: 0.158, 0.126, 0.08 vs 0.427, respectively). Throughout the OLE, the difference in volume accumulated in the DBP was largely maintained, despite the switching of patients from the comparator arm to ocrelizumab. After ~7 years, ocrelizumab patients still showed 16% less thalamic volume loss compared with IFNβ1a–ocrelizumab patients in OPERA, and 26% less compared with placebo–ocrelizumab patients in ORATORIO (Figure 2). When OLE trajectories of the thalamic volume decrease in RMS were compared, ocrelizumab and IFNβ1a–ocrelizumab patients showed similar slopes (*p* = 0.124). Considering timepoints occurring after OLE week 46 (to avoid confounding due to the reversal of pseudoatrophy in patients switching from IFNβ1a), estimated yearly percentage changes for IFNβ1a–ocrelizumab and ocrelizumab were −0.33% (95% confidence interval (CI): −0.35% to −0.30%) and −0.38 % (95% CI: −0.40% to −0.35%) per year, respectively. In PPMS, placebo–ocrelizumab had a slightly lower rate of volume loss than ocrelizumab, but it was not significant (*p* = 0.071). When considering timepoints after OLE day 1, estimated yearly percentage changes for placebo–ocrelizumab and ocrelizumab were −0.42% (95% CI: −0.48% to −0.37%) and −0.53 % (95% CI: −0.57% to −0.49%) per year, respectively. Especially in PPMS, a negative correlation between the intercept (thalamic volume at the first timepoint considered) and the slope (rate of thalamic volume loss) was observed, indicating that placebo–ocrelizumab showed slightly less thalamic volume loss because of lower thalamic volume after the DBP as a starting point. Baseline T2 lesion volume showed weak-to-moderate correlations with thalamic volume loss at the end of the DBP, with higher associations found for the comparator arms (correlation coefficient, *p* value; RMS, IFNβ1a: −0.296, *p* < 0.001, ocrelizumab: −0.209, *p* < 0.001; PPMS, placebo: −0.377, *p* < 0.001, ocrelizumab: −0.231, *p* < 0.001). When considering T2 lesion volume changes, instead, no correlation was found in RMS in either arm (*p* > 0.05), while there was a weak correlation in PPMS and more pronounced in the placebo arm (correlation coefficient, *p* value; placebo: −0.27, *p* < 0.001; ocrelizumab: −0.13, *p* = 0.021).

### RMS versus PPMS comparison

Patients with RMS and PPMS had similar baseline thalamic volumes, after adjusting for baseline characteristics (adjusted mean: RMS: 14.92 ± 1.73 cm^3^; PPMS: 14.83 ± 0.58 cm^3^; *p* = 0.289) as well as similar thalamic volume loss rates when treated with ocrelizumab (*p* = 0.481). In particular, estimated yearly percentage changes for OPERA and ORATORIO were −0.45% (95% CI: −0.47% to −0.42%) and −0.46% (95%CI: −0.49% to −0.42%) per year, respectively.

### Baseline thalamic volume and clinical outcomes association

Lower thalamic volume at baseline was significantly associated with the 9HPT, T25FW, and EDSS score in RMS and PPMS (Table 2).

**Table 2.** Association between baseline thalamic volume and baseline 9HPT, EDSS, and T25FW in RMS and PPMS populations.

	RMS (OPERA I/II)			PPMS (ORATORIO)		
	9HPT (s)	EDSS	T25FW (s)	9HPT (s)	EDSS	T25FW (s)
Estimates (standard error), *p* value, cm^3^	–1.075 (0.185), *p* < 0.001	–0.163 (0.020), *p* < 0.001	–0.461 (0.168), *p* = 0.006	–2.587 (0.530), *p* < 0.001	–0.107 (0.031), *p* < 0.001	–1.933 (0.518), *p* < 0.001

9HPT: Nine-Hole Peg Test; EDSS: Expanded Disability Status Scale; T25FW: Timed 25-Foot Walk Test; PPMS: primary progressive multiple sclerosis; RMS: relapsing multiple sclerosis; s: seconds.

For disability progression occurring during the DBP, in RMS, the interaction between baseline thalamic volume and treatment was significant for CCDP12 (*p* = 0.019), CCDP24 (*p* = 0.024), CDP12-9HPT (*p* = 0.004), and CDP24-9HPT (*p* = 0.007). When the treatment arms were considered separately, baseline thalamic volume was associated with increased risk of progression in the IFNβ1a-treated patients, but not for those randomized to ocrelizumab (Table 3). For the other disability progression measures, there was no evidence of an association between baseline thalamic volume and treatment effect on disability progression occurring during DBP (*p* > 0.05 for all models) (Supplemental Table S1). In PPMS, baseline thalamic volume was not significantly associated with any progression measures (*p* > 0.05 for all models) (Supplemental Table S2).

**Table 3.** Association between baseline thalamic volume and 24-week confirmed disability progression measured by 9HPT and CCDP in the IFNβ1a and the ocrelizumab treatment arms, in the RMS population (multiple Cox regression).

	CDP24-9HPT^a^		CCDP24^b^	
	IFNβ1a	Ocrelizumab	IFNβ1a	Ocrelizumab
Events, *n* (%)	35 (4.23)	25 (3.02)	205 (24.76)	154 (18.62)
Baseline thalamic volume (cm^3^), HR [95% CI], *p* value	0.79 [0.64–0.98], 0.031	1.22 [0.91–1.64], 0.192	0.87 [0.80–0.95], 0.003	0.96 [0.86–1.08], 0.531

9HPT: Nine-Hole Peg Test; CCDP24: composite confirmed disability progression at 24 weeks; CDP24: confirmed disability progression at 24 weeks; CI: confidence interval; HR: hazard ratio; IFNβ1a: interferon β-1a.

a Time to CDP24 measured by an increase from double-blind baseline in the time to complete 9HPT of ⩾20%.

b Time to CCDP24 measured by an increase from double-blind baseline in EDSS score of least 1.0 point (or 0.5 points for a baseline score above 5.5), the time to perform the T25FW of ⩾20%, or the time to complete 9HPT of ⩾20%.

## Discussion

We assessed thalamic volume changes in patients with RMS and PPMS, quantified the effect of an anti-CD20 therapy on thalamic volume loss in these populations, and evaluated the usefulness of baseline thalamic volume as a potential prognostic/predictive tool to capture the risk of subsequent disability progression.

Ocrelizumab significantly reduced thalamic volume loss in patients with RMS and PPMS, and the advantage acquired during the DBP was maintained during the OLE when the comparator arm switched to ocrelizumab. Previously, Sotirchos et al.^25^ showed patients in an observational cohort on high-efficacy DMTs (natalizumab or rituximab) had a reduced annualized percentage change in thalamic volume loss compared with low-efficacy DMTs (interferon-beta or glatiramer acetate). However, the groups were not randomized and rituximab-specific results were not reported.^25^ Thus, to our knowledge, this is the first work that looks at a large, randomized population of patients with RMS and PPMS to assess the effect of an anti-CD20 treatment on thalamic volume.

Ocrelizumab showed the greatest effect on thalamic volume compared with whole brain, white or cortical gray matter. This could be explained from the perspective that thalamic injury may reflect much of the MS-related damage that occurs throughout the whole CNS and may not be specific to the thalamus only.^26^ For instance, demyelinating lesions can directly affect the thalamus;^1^ however, thalamic lesion volume does not seem to correlate with thalamic volume loss.^27^ It seems that lesions outside the thalamus play a more important role as evidenced by the relation of thalamic volume loss to white and gray matter lesions in general,^5,27^ and to lesions in thalamocortical projections, in particular.^28^ The importance of white matter lesions is further supported by the strong negative correlation we found between baseline T2 lesion volume and thalamic volume as well as the weak-to-moderate associations with thalamic volume loss at the end of the DBP, especially in the comparator arms. However, no association or only a weak association was found between thalamic volume loss and T2 lesion volume changes at the end of the DBP in RMS and PPMS, respectively. We could speculate that concurrent acute inflammation in the white matter, more predominant in RMS, had less direct impact on reducing the thalamic volume, while secondary neurodegeneration due to T2 lesions occurs more gradually, requiring years before appreciating an effect on thalamic volume loss. Moreover, not only lesions, but other pathological mechanisms could affect the thalamus. Postmortem histopathology studies connected the presence of thalamocortical tract-specific pathology, that is, myelin loss in non-lesional white matter, with the neurodegeneration of cortical and thalamic gray matter regions,^29^ as well as neuronal and thalamic volume loss.^5^

It remains unclear whether other mechanisms might explain the difference between the treatment arms. For example, it is unknown whether ocrelizumab could create a milieu in which remyelination and tissue repair can be promoted, as suggested by the work by Vavasour et al.,^30^ showing increased myelin water fraction in “normal appearing” white matter corpus callosum and corticospinal tract of ocrelizumab-treated patients.

Independently from causes of thalamic volume loss, our results suggest common mechanisms between ocrelizumab-treated patients with RMS and PPMS that result in similar rates of thalamic volume loss, when new focal inflammation and relapses are essentially abrogated. Regardless of common mechanisms, a treatment effect is evident from week 24 in RMS, but only from week 120 in PPMS, a difference most likely driven by the pseudoatrophy affecting patients treated with IFNβ1a^31,32^ and partially reversible when switching to ocrelizumab. Those common mechanisms seem to be irreversible, despite the use of high-efficacy therapy, underscoring the need to treat patients with MS with high-efficacy DMTs earlier to preserve brain tissue.

At baseline, thalamic volume was associated with EDSS, 9HPT, and T25FW in patients with RMS and PPMS, consistent with previous reports.^28,33,34^ Baseline thalamic volume might also predict future treatment effect on disability progression as measured by 9HPT and CCDP occurring during the DBP in RMS. Across treatment groups, we found that a higher thalamic volume at baseline was associated with a decrease in progression events (measured by 9HPT and CCDP) in the IFNβ1a arm, but not in the ocrelizumab arm (Supplemental Table S1). For PPMS, instead, no association was seen between baseline thalamic volume and future risk of disability progression (measured by EDSS, 9HPT, T25FW, or CCDP). This may suggest that therapies with a high anti-inflammatory effect could favorably change the progression trajectory and the relationship with MRI measures in RMS, but not in PPMS, where perhaps other mechanisms, more chronic and with a less direct impact, are more pronounced.

In a previous work, Eshagi et al.^34^ found a connection between baseline thalamic volume and future EDSS changes. Others observed an association with EDSS worsening after 5 years with fractional anisotropy of the thalamus, but not with thalamic volume.^35^ The low number of progression events and different definitions of progression may underlie the discrepancy with our results.

Our study has limitations. We did not measure thalamic lesions that could allow for the discrimination between direct and indirect effects on volume change. Inflammation within the deep gray matter is less intense, generating a lower contrast between affected and non-affected deep gray matter than inflammation in the white matter, making it difficult to appreciate in conventional MRI. Moreover, the RMS placebo group could have helped in further understanding the initial accelerated thalamic volume decrease in the IFNβ1a arm.

In conclusion, our results show that ocrelizumab effectively reduces thalamic volume loss in patients with RMS and PPMS, with the highest effect size compared with whole brain, white and cortical gray matter. Moreover, ocrelizumab helps preserve thalamic volume when started earlier. These findings, together with the association between thalamic volume and disability, suggest that measurement of thalamic volume loss may be a particularly useful biomarker for assessing treatment effects on the prevention of tissue damage.

## Supplemental Material

sj-docx-1-msj-10.1177_13524585221097561 – Supplemental material for Ocrelizumab reduces thalamic volume loss in patients with RMS and PPMS Click here for additional data file. Supplemental material, sj-docx-1-msj-10.1177_13524585221097561 for Ocrelizumab reduces thalamic volume loss in patients with RMS and PPMS by Douglas L Arnold, Till Sprenger, Amit Bar-Or, Jerry S Wolinsky, Ludwig Kappos, Shannon Kolind, Ulrike Bonati, Stefano Magon, Johan van Beek, Harold Koendgen, Oscar Bortolami, Corrado Bernasconi, Laura Gaetano and Anthony Traboulsee in Multiple Sclerosis Journal
