# Supplementary material for: Ocrelizumab reduces thalamic volume loss in patients with RMS and PPMS
Source: Mult Scler. 2022 Jun 7;28(12):1927–36. doi: 10.1177/13524585221097561 (PMC9493406; doi:10.1177/13524585221097561)
Supplement: sj-docx-1-msj-10.1177_13524585221097561 – Supplemental material for Ocrelizumab reduces thalamic volume loss in patients with RMS and PPMS [file sj-docx-1-msj-10.1177_13524585221097561.docx]

**Supplementary materials**

**Table S1:** Association between baseline thalamic volume and disability progression occurring during the DBP in RMS when interaction between baseline thalamic volume and treatment is not considered.

|  | **CDP12-9HPT^a^** | | **CDP24-9HPT^a^** | | **CDP12-EDSS^b^** | | **CDP24-EDSS^b^** | | **CDP12-T25FW^c^** | | **CDP24-T25FW^c^** | | **CCDP12^d^** | | **CCDP24^d^** | |
| --- | --- | --- | --- | --- | --- | --- | --- | --- | --- | --- | --- | --- | --- | --- | --- | --- |
|  | **IFNβ1a** | **OCR** | **IFNβ1a** | **OCR** | **IFNβ1a** | **OCR** | **IFNβ1a** | **OCR** | **IFNβ1a** | **OCR** | **IFNβ1a** | **OCR** | **IFNβ1a** | **OCR** | **IFNβ1a** | **OCR** |
| **Events, n (%)** | 42 (5.07) | 33 (3.99) | 35 (4.23) | 25 (3.02) | 123 (14.86) | 82 (9.92) | 104 (12.56) | 67 (8.10) | 147 (17.75) | 115 (13.91) | 116 (14.01) | 92 (11.12) | 249 (30.07) | 183 (22.13) | 205 (24.76) | 154 (18.62) |
| **Baseline thalamic volume (cm^3^), HR (95%CI), p value** | 0.88 (0.75–1.03), 0.106 | | 0.91 (0.77–1.09), 0.301 | | 0.91 (0.83–1.00), 0.061 | | 0.95 (0.86–1.06), 0.361 | | 0.95 (0.88–1.04), 0.252 | | 0.93 (0.85–1.02), 0.133 | | 0.90 (0.84–0.96), 0.001 | | 0.90 (0.84–0.97), 0.005 | |

9HPT, Nine-Hole Peg Test; CCDP, composite confirmed disability progression; CDP, confirmed disability progression; CI, confidence interval; EDSS, expanded disability status scale; HR, hazard ratio; IFNβ1a, interferon β-1a; n, number; OCR, ocrelizumab; T25FW, Timed Twenty-Five Foot Walk.

^a^Time to CDP12 or CDP24 measured by an increase from double-blind baseline in the time to complete 9HPT of ≥20%.

^b^Time to CDP12 or CDP24 measured by an increase from double-blind baseline in EDSS score of least 1.0 point (or 0.5 points for a baseline score above 5.5).

^c^Time to CDP12 or CDP24 measured by an increase from double-blind baseline in the time to complete T25FW of ≥20%.

^d^Time to 12-week or 24-week confirmed occurrenceof an increase in EDSS score, the time to perform the T25FW of ≥20%, or the time to complete 9HPT of ≥20%.

**Table S2:** Association between baseline thalamic volume and disability progression occurring during the DBP in PPMS when interaction between baseline thalamic volume and treatment is not considered.

|  | **CDP12-9HPT^a^** | | **CDP24-9HPT^a^** | | **CDP12-EDSS^b^** | | **CDP24-EDSS^b^** | | **CDP12-T25FW^c^** | | **CDP24-T25FW^c^** | | **CCDP12^d^** | | **CCDP24^d^** | |
| --- | --- | --- | --- | --- | --- | --- | --- | --- | --- | --- | --- | --- | --- | --- | --- | --- |
|  | **PBO** | **OCR** | **PBO** | **OCR** | **PBO** | **OCR** | **PBO** | **OCR** | **PBO** | **OCR** | **PBO** | **OCR** | **PBO** | **OCR** | **PBO** | **OCR** |
| **Events, n (%)** | 52 (21.31) | 68 (13.96) | 47 (19.26) | 60 (12.32) | 80 (32.79) | 138 (28.34) | 76 (31.15) | 129 (26.49) | 131 (53.69) | 212 (43.53) | 120 (49.18) | 185 (37.99) | 156 (63.93) | 258 (52.98) | 147 (60.25) | 232 (47.64) |
| **Baseline thalamic volume (cm^3^), HR (95%CI), p value** | 0.97 (0.85–1.11), 0.642 | | 0.96 (0.83–1.10), 0.545 | | 1.03 (0.93–1.13), 0.595 | | 1.05 (0.95–1.16), 0.351 | | 1.05 (0.97–1.13), 0.243 | | 1.07 (0.99–1.16), 0.102 | | 1.05 (0.97–1.12), 0.225 | | 1.06 (0.98–1.41), 0.126 | |

9HPT, Nine-Hole Peg Test; CCDP, composite confirmed disability progression; CDP, confirmed disability progression; CI, confidence interval; EDSS, expanded disability status scale; HR, hazard ratio; PBO, placebo; n, number; OCR, ocrelizumab; T25FW, Timed Twenty-Five Foot Walk.

^a^Time to CDP12 or CDP24 measured by an increase from double-blind baseline in the time to complete 9HPT of ≥20%.

^b^Time to CDP12 or CDP24 measured by an increase from double-blind baseline in EDSS score of least 1.0 point (or 0.5 points for a baseline score above 5.5).

^c^Time to CDP12 or CDP24 measured by an increase from double-blind baseline in the time to complete T25FW of ≥20%.

^d^Time to 12 week or 24 week confirmed occurrence of an increase in EDSS score, the time to perform the T25FW of ≥20%, or the time to complete 9HPT of ≥20%.
